# Perceptions of Emerging Adults With Type 1 Diabetes Mellitus on How the Condition Influences Sleep Quality: A Qualitative Study

**DOI:** 10.1155/2024/7497059

**Published:** 2024-07-10

**Authors:** María-Ángeles Núñez-Baila, Anjhara Gómez-Aragón, José Rafael González-López

**Affiliations:** ^1^ Nursing Department Faculty of Nursing Physiotherapy and Podiatry Universidad de Sevilla, Seville, Spain; ^2^ Instituto de Biomedicina de Sevilla IBiS/Hospital Universitario Virgen del Rocío/CSIC/Universidad de Sevilla, Seville, Spain

**Keywords:** adult, diabetes mellitus type 1, perception, qualitative research, sleep, young adult

## Abstract

**Background:** Emerging adulthood is a phase characterized by exploration which potentially affecting sleep quality. While many emerging adults are healthy, the effects of chronic diseases such as Type 1 Diabetes Mellitus (T1DM) on sleep may be underestimated. Considering the frequency of nocturnal glycemic alterations that cause awakenings, this study explored the perceptions of emerging adults in Andalusia on the influence of T1DM on their sleep quality.

**Methods:** A qualitative approach was used for this study. Purposive sampling through diabetes associations was initially utilized, supplemented by snowball sampling, in order to conduct semistructured interviews with 73 emerging adults (aged 18–29) diagnosed with T1DM, to explore their perceptions of the influence of T1DM on sleep quality. Interpretative Phenomenological Analysis was used for data analysis.

**Results:** Sleep disruptions caused by overnight hyperglycemia and hypoglycemia were identified as significant factors. However, 62% of participants did not perceive the influence of diabetes on their sleep quality, despite experiencing frequent overnight glycemic alterations (reported by 40.9%).

**Conclusions:** Perception of the impact of T1DM on sleep quality does not always align with the frequency of disruptions. Nonetheless, promoting healthy sleep and systematically assessing sleep quality can benefit both sleep and glycemic outcomes, regardless of individual perceptions.

## 1. Introduction

Emerging adulthood is a transition stage spanning between ages 18 and 29 in which young people commonly explore myriad life spheres such as social and sexual relationships or substance use [1]. New challenges such as flat sharing, part-time jobs, or a high demand of social interaction stimulate nightlife activity, therefore affecting their sleep pattern [2–4].

Starting from adolescence, the desire for increased autonomy and the need of meeting academic, work, and social expectations may impact on sleep quality by altering weekly times and schedules on working days and over the weekend, therefore shortening sleep duration and generating daytime sleepiness. During emerging adulthood, this pattern continues showing very similar characteristics to those of adolescence, with a high probability of underestimating the consequences of altering sleep patterns and experiencing social jet lag [3, 5]. All these factors have a greater impact in emerging adults with Type 1 Diabetes Mellitus (T1DM), as they need to balance self-care requirements with those of the academic, work, and social spheres [6, 7]. A poor management of this situation may result in overnight hyperglycemia or hypoglycemia episodes which, in turn, affect sleep [8, 9].

Quantitative research has proved that people with T1DM had worse sleep quality in comparison to those people without T1DM [10]. High levels of hemoglobin A1c (HbA1c) are associated to shortened sleep and affect both academic and work performance [11]. Poor sleep involves long daily hypoglycemic symptoms after returning to normoglycemia, longer cognitive dysfunction, and lower insulin sensitivity [12–14]. Sleep deficiency is also associated with poor executive function, memory loss, and behavioural dysregulation, therefore affecting the essential skills to manage T1DM [3, 10, 15]. The relation between glycemic variability and sleep is bidirectional: high levels of glycemia are associated to lower levels of melatonin in adults with T1DM [16]. At the same time, poor sleep quality, including high sleep fragmentation, affects glycemic stability which commonly reaches higher HbA1c values, 53 mmol/mol (7%) or above [16]. Quantitative research has revealed a significant association between overnight glycemic variability, including both hyperglycemia and hypoglycemia, and the sleep quality across various age stages, but with limited focus on emerging adulthood. Additionally, it is important to highlight that the frequency of these glycemic alterations has not been consistently reported in the literature [12, 15, 17]. Furthermore, despite the established correlation between nocturnal glycemic alterations and quality of life, there still exists an analytical gap in considering that individuals who experience a high frequency of these alterations and do not show poor sleep quality [10]. Therefore, further investigation is necessary to comprehensively understand the behavioural mechanisms involved. Conducting qualitative studies that focus on individuals' perceptions can offer an initial approach to shed light on these mechanisms [18, 19].

Regarding qualitative investigations, some barriers and facilitators have been identified, such as the fear of nocturnal hypoglycemia, alarms from diabetes devices (both real and false), and overnight hyperglycemia or hypoglycemia. These factors, along with their consequences, have been reported by individuals with T1DM and their partners and families [17, 18]. On the contrary, on many occasions, the relationship between sleep quality and T1DM has primarily emerged incidentally during discussions on other aspects regarding quality of life, resulting in potential gaps due to the absence of premeditated research questions [19]. Moreover, the adolescent stage has received more extensive research attention compared to other stages. For example, in the qualitative study by Bergner et al. [20], adolescents with T1DM identified screen use before going to the bed as the primary barrier to sleep. In contrast, their parents highlighted nocturnal glycemic alterations as the main factor interrupting their sleep. However, the underlying reasons for these divergent perceptions and the frequency of these alterations were not specifically examined. The American Diabetes Association (ADA) [21] recommends the assessment of sleep pattern in people with T1DM. However, as there are no specific indications to perform it, there is a paucity of studies focusing on this field [10]. Regarding the stage of emerging adulthood, the limited body of qualitative research indicates that the approach has varied. For instance, Griggs et al. [18] examined the self-reported objectives of emerging adults with T1DM related to diabetes and sleep quality, revealing management of glycemic levels during the night to enhance sleep quality as one of them. This area is particularly interesting and understudied within this age group. In this line, the study conducted by Griggs et al. [17] explored barriers and facilitators related to diabetes with respect to sleep, where overnight hyperglycemia and hypoglycemia episodes were recognized by all participating emerging adults with T1DM. However, the participants' perception of these nocturnal glycemic alterations was not compared with a frequency reference. For this reason, considering the frequency of nocturnal glycemic alterations that cause awakenings, this study is aimed at exploring the perceptions of emerging adults in Andalusia on the influence of T1DM on their sleep quality.

## 2. Materials and Methods

### 2.1. Study Design

This study, through a qualitative design and grounded in Interpretative Phenomenological Analysis (IPA) [22], explores how emerging adults in Andalusia perceive the influence of T1DM on their sleep quality, considering the frequency of nocturnal glycemic alterations that cause awakenings.

### 2.2. Setting and Respondents

In this study, in-depth, semistructured, personal interviews were conducted. Pilot interviews were carried out in two phases: One interview took place in February 2020, before the COVID-19 lockdown, and the remaining eight interviews were conducted in August 2020, following the lockdown. The data from these interviews were included in the analysis, subject to an evaluation of the relevance of the information gathered. Following that, the work camp continued with 64 interviews conducted between November 2021 and April 2022. This qualitative study is a part of a larger research project that analyzes the quality of life and lifestyles of emerging adults with T1DM. Furthermore, the researchers are part of the research group named *CTS284: Promoción de la Salud* addressing the lifestyle of emerging adults with T1DM, including sleep habits. A semistructured approach was employed during the interviews. Dichotomous questions were used when the responses from the open-ended question did not provide a clear answer that aligned with our objectives. However, it is essential to emphasize that after each dichotomous question, open-ended question was addressed to participants to encourage them to elaborate on their previous responses by providing explanations and reasons. A topic guide on T1DM and sleep quality (supplementary material (available here)) provided a rich data amount for independent analysis. Parts of the interviews addressing diabetes and sleep quality varied in duration, with a median of 10 min and 56 s and a range being from 3 min and 27 s to 31 min and 4 s. Participants' interviews were recorded using the Samsung Galaxy Table S7® tablet voice recording application. Each interview saved as an independent audio file. Nonverbal communication was also noted down. This study includes emerging adults (aged 18–29) with T1DM for at least 1 year and living in Andalusia (Spain). Psychiatric disorders and speech/listening difficulties which may interfere with interviews were considered exclusion criteria.

Sample was recruited with the help of 13 of the 55 diabetes associations in Andalusia, where the main researcher intentionally contacted these associations to offer a free workshop to recruit attendees, employing a purposive strategy to initially engage participants. Furthermore, as previously described, this research is part of a larger project. At the end of the quantitative survey of the project, participants were given a link to a separate, nonidentifiable questionnaire for independent participation in the interview of the qualitative study. Participants were contacted via phone calls and WhatsApp messages, as these were the preferred contact methods. Due to their lack of time and the uprooting of emerging adults with T1DM regarding associationism, the snowball effect technique was subsequently used to increase the number of participants.

MANB (the main researcher, a female nurse and PhD student sharing the participants' age range) accomplished the processes of contact, recruitment, and interviews. This researcher had previous experience in conducting semistructured interviews in this population [23], and she was already sensitized due to her participation in camps for children and adolescents with diabetes. All this facilitated a closer communication and an atmosphere of trust.

### 2.3. Data Analysis

Sociodemographic data were analyzed with SPSS v26® software. For the qualitative data, the subsequent steps were accomplished, following the IPA guidelines [22]: First, audio recordings of the interviews were transcribed verbatim and imported into NVivo v12™ software for organization. The first three transcripts were then read multiple times by the main and second researchers (MANB and AGA) to immerse themselves in the data, a key step in IPA to comprehensively understanding the content. Initial notes were made on these readings, focusing on descriptive, linguistic, and conceptual comments which are crucial for identifying emergent themes in IPA. These initial notes were then transformed into emergent themes, through an iterative process of engagement with the data. MANB and AGA independently coded the first three interviews, identifying four superordinate themes by aggregation and ten emergent themes, detailed in Table 1. No more interviews were coded in the initial steps because no new superordinate or emergent themes were found after the third interview. This independent analysis ensures that researchers engage deeply with the data from their unique perspectives, another core principle of IPA. This rigorous approach resulted in an intercoder reliability rate of 79%. Therefore, this consensus coding was used as a guide to classify the data from the remaining interviews, which subsequently were coded by the main researcher. When discourse was not clear enough, its interpretation was verified with the participants involved. This verification step is in line with the emphasis of IPA on validating findings with participants whenever possible.

Refining our data analysis, it was observed that participants' responses to the superordinate theme of perception of sleep linked with the responses of each of the remaining three superordinate themes (overnight glycemic management: hypoglycemia, overnight glycemic management: hyperglycemia, and effects of overnight glycemic alterations). In aligning our qualitative analysis with the frequency of overnight glycemic alterations that cause awakenings, we adhered to the diagnostic criteria for sleep disorders as compiled in the fifth edition of the Diagnostic and Statistical Manual of Mental Disorders (DSM-5) [24]. The DSM-5 stipulates that experiencing awakening at night on 3 days per week serves as a benchmark [24]. Accordingly, the cut-off points used in this study were ≤ 2 and > 2 days to classify the number of awakenings per week generated by overnight glycemic alterations.

### 2.4. Ethical Considerations

This study adheres to The Code of Ethics of the World Medical Association for experiments involving humans (Declaration of Helsinki). Additionally, this study, with the code 2150-M1-22, was approved by the Ethics Committee of the University Hospitals Virgen Macarena and Virgen del Rocío in Seville, Spain, on February 11, 2020. Before each interview, participants gave their written consent and were informed about the object of the research, who the researcher was, and the motives to conduct the study. All interviews were conducted solely between the researcher and the interviewee, either at the participant's home or a facility hired by the researcher-interviewer.

Confidentiality was guaranteed through the codification of verbatims indicating the number of interview, sex, age, HbA1c value, and type of treatment (multiple daily injections [MDIs] or continuous subcutaneous insulin infusion [CSII]) in the following format: (E1, woman 25, HbA1c 53 mmol/mol (7%), MDI).

## 3. Results

### 3.1. Sample Characteristics and Identified Themes

This study engaged 73 participants; however, two were excluded due to insufficient information regarding sleep. Ultimately, the sample consisted of 71 participants (57.7% female) with a median age of 20 years, ranging from 18 to 29 years. The median of diabetes duration among the participants was 11 years, ranging from 2 to 27 years. Regarding treatment, 73.2% of participants used MDIs and 26.8% CSII. More than half (65.8%) displayed HbA1c levels above 53 mmol/mol (7%). Sample characteristics are detailed on Tables 2 and 3.

Table 4 illustrates participants' responses to the superordinate theme *perception of sleep* linked with the responses of each of the remaining three superordinate themes (*overnight glycemic management: hypoglycemia*, *overnight glycemic management: hyperglycemia*, and *effects of overnight glycemic alterations*).

### 3.2. T1DM and Sleep Quality

Almost half of our 71 participants (47.9%) experience three or more glycemic alterations per week causing awakening. After an overnight glycemic alteration, 35.2% of total emerging adults with T1DM interviewed declared to be awake for thirty minutes or more, and 38% experienced difficulty in getting back to sleep. Moreover, 69% of total interviewees perceived negative consequences at academic, work, social, or personal levels the day after an episode of sleep disruption due to overnight glycemic alterations.

#### 3.2.1. People Who Perceive the Influence of T1DM on Their Sleep Quality

When participants were asked about the influence of diabetes on sleep quality, it was found that 38% (27 out of 71) declares to perceive the influence of T1DM on sleep quality. Of this total, 44.4% (12 out of 27) declares to perceive it *many times*, and 55.6% (15 out of 27) only *sometimes*. This means that more than one-third of the interviewees explicitly state this influence: *“Clearly, because sleep gets interrupted for this or that; or you need to go to the bathroom, or eat, or if, for example, the insulin pump starts to beep, or this* [points out the mobile phone] *vibrates to tell you something is happening… Of course it influences sleep*. *This is not just a careless sleep in which you just worry about breathing, you know? In my opinion, yes, there's a strong influence.”* (E18, Woman 22, HbA1c 54 mmol/mol (7.1%), MDI).

##### 3.2.1.1. Frequency of Overnight Glycemic Alterations

Among the participants, 59.3% (16 out of 27) perceive this influence and experience three or more overnight glycemic alterations per week. Notwithstanding, perception of T1DM influencing sleep quality *many times* may be also associated with experiencing two or less overnight glycemic alterations per week: *“Yes, I'd say there are a lot. Hyperglycemias, hypoglycemias, pump alarms… yes, definitely, yes.”* (E70, Woman 24, HbA1c 67 mmol/mol (8.3%), CSII). Likewise, the number of overnight glycemic alterations and span of interrupted sleep in the same night are other influencing factors: *“Those nights* [experiencing hyperglycemia] *I've to wake up five or six times to go to the bathroom, even if I've had my insulin dose and levels are getting low. The first time I got asleep very quickly, but after that one, I need twenty minutes at least to go back to sleep.”* (E17, Man 20, HbA1c 55 mmol/mol (7.2%), MDI).

##### 3.2.1.2. Negative Effects Perceived on the Next Day After Overnight Glycemic Alterations

Negative effects at academic, work, social, or personal levels are experienced by 92.6% of the interviewees who perceive the influence of T1DM on sleep quality (25 out of 27): *“For example, in my case, now that you have mentioned the examinations* [for public servants], *as I said before, I've been sleeping awfully for months now, because of the hypoglycemias. So, when I wake up in the morning, it's like: ‘Oh, my god, I've to study'. I feel terribly tired and sleepy every day and I think that maybe that's different for someone not having this* [diabetes]*.”* (E58, Woman 26, HbA1c 41 mmol/mol (5.9%), MDI). Even when they have two or less overnight glycemic alterations, they may still perceive relevant negative effects the following day: *“On the next day* [after a hyperglycemia episode] *I feel absolutely knackered, ha, ha!”* (E44, Man 19, HbA1c 70 mmol/mol (8.6%), MDI).

##### 3.2.1.3. Difficulty in Getting Back to Sleep After Overnight Glycemic Alterations and Span of Sleep Interruption

Of the participants perceiving the influence of T1DM on their sleep, 48.2% (13 out of 27) expressed difficulty in getting back to sleep after an overnight glycemic alteration: *“Obviously, yes. If your sugar levels are low before falling asleep, at least in my case, it's hard to fall asleep if I do not have it under control first.”* (E67, Man 20, HbA1c 46 mmol/mol (6.4%), MDI). It is relevant that 44.4% of emerging adults perceiving the influence of T1DM on sleep quality (12 out of 27) experience a sleep interruption of more than thirty minutes after an overnight glycemic alteration: *“It takes around 30 to 45 minutes, as much.”* (E55, Man 29, HbA1c 50 mmol/mol (6.7%), MDI). This would mean that almost half of the participants perceiving the influence of diabetes on sleep quality also declare to experience difficulties to getting back to sleep after interruptions lasting more than thirty minutes.

##### 3.2.1.4. Other Factors Affecting the Perception of the Influence of T1DM on Sleep Quality

The precise moment in which the glycemic alteration occurs during the night is key: *“Depending on the time I have it…* [hypoglycemia episode] *If it's 5 am, yes* [it does affect her]*, in fact, I do not want to go to class, I say to myself ‘It's just that I've not rested'. Because when I notice low at night* [overnight hypoglycemia]*, maybe I've been like that for three or four hours* [overnight hypoglycemia]*, and then you think, hey, I'm weary, I do not want to go anywhere, I just want to sleep, at least for the time wasted due to the sugar drop.”* (E15, Woman 20, HbA1c 64 mmol/mol (8%), MDI). Diabetes devices, which help to optimize glycemic management, also influence sleep quality. Specifically, the continuous glucose monitoring devices or CSII endowed with alarm systems, when detecting and episode of hyperglycemia or hypoglycemia, may awake the sleeping owner with their alerting sound: *“What happens many times is that it wakes me up like five times in one night. And that is… whether you like it or not… it's a bummer, because it* [the Continuous Subcutaneous Insulin Infusion alarm] *does not alert just once to tell you your levels have dropped, but one time like ‘It's going down* [glycemia levels]*, the pump will stop', then another like ‘It's going up* [glycemia levels]*', and then another one telling you ‘The basal* [insulin] *it's going to be adjusted'*. *And all this just for one drop* [overnight hypoglycemia]*, if you have more than one, this takes like forever.”* (E1, Woman 21, HbA1c 60 mmol/mol (7.6%), CSII).

#### 3.2.2. People Who Do Not Perceive the Influence of T1DM on Sleep Quality

To the same question, 62% of total participants (44 out of 71) declared not perceiving the influence of T1DM on their sleep quality. Of this total, 25% (11 out of 44) pointed out that *rarely* perceived this influence and 75% (33 out of 44) affirmed *never* to perceived it. Therefore, more than half of participants do not perceive that diabetes influences their sleep quality: *“No, I think it does not. I think I sleep well.”* (E56, Man 22, HbA1c 57 mmol/mol (7.4%), CSII).

##### 3.2.2.1. Frequency of Overnight Glycemic Alterations

Among those participants who do not perceive the influence of T1DM on sleep, 40.9% (18 out of 44) experience three or more glycemic alterations at night. Even though more than half of participants who do not perceive the influence of T1DM (59.1%, 26 out of 44) declares two or less overnight hypoglycemia or hyperglycemia episodes per week, the decisive role of overnight glycemic alterations on sleep quality is acknowledged: *“I think that a well-controlled diabetes does not influence, but a poor management does. Right now, I'd say that my sleep quality is good. If it* [glycemia] *wasn't controlled, it'd probably affect my sleep a lot.”* (E68, Man 24, HbA1c 46 mmol/mol (6.4%), MDI). However, having three or more overnight glycemic alterations every week may be not considered a high frequency by emerging adults who were interviewed, as, for example, this case, a participant who declared to experience up to three hypoglycemias in one week: *“No. No, because I do not have too many drops and stuff.”* (E9, Woman 18, HbA1c 56 mmol/mol (7.3%), MDI).

##### 3.2.2.2. Negative Effects Perceived on the Next Day After Overnight Glycemic Alterations

Among the participants who did not perceive the influence of T1DM on sleep quality, 45.5% (20 out of 44) declared perceiving no negative effects at academic, work, social, or personal levels the day after experiencing an overnight glycemic alteration, though 40% of them (8 out of 20) has three or more overnight glycemic alterations per week. This means that, despite more than a third of the interviewees who do not perceive the influence of T1DM on sleep quality declared to experience three or more overnight glycemic alterations per week, these may not be associated to negative effects on the following day: *“No. Sometimes I feel more tired, but not because it* [hypoglycemia] *wakes me up, but because maybe I've slept worse.”* (E57, Man 22, HbA1c 55 mmol/mol (7.2%), CSII).

##### 3.2.2.3. Difficulty in Getting Back to Sleep After Overnight Glycemic Alterations and Span of Sleep Interruption

Among the interviewees who expressed that T1DM does not influence their sleep quality, 68.2% (30 out of 44) also declared not having any difficulty in going back to sleep after an overnight glycemic alteration. However, 46.7% (14 out of 30) of those who reported not having this difficulty declared having three or more overnight glycemic alterations per week. This means that a high frequency of overnight hyperglycemia or hypoglycemia causing awakening does not determine the difficulty of getting back to sleep. This situation was expressed as a routine: *“No, I just wake up, test it* [glucose test], *adjust my insulin and go back to sleep. I mean, I do not stay awake thinking or having trouble getting back to sleep. I just fall sleep and that's all.”* (E24, Woman 21, HbA1c 48 mmol/mol (6.5%), MDI). Likewise, 29.5% (13 out of 44) of participants who do not perceive the influence of T1DM on sleep quality experience a sleep interruption of thirty minutes or more after an overnight glycemic alteration, though this may be not relevant for the interviewees: “*No, it does not* [the person declares that diabetes does not influence sleep]*. Because I feel fine the next day. I do not feel like super tired because I have not slept for half an hour, no. I wake up OK.”* (E13, Woman 20, HbA1c 53 mmol/mol (7%), MDI).

##### 3.2.2.4. Other Factors Affecting the Perception of the Influence of T1DM on Sleep Quality

In this age group, other factors such as stress may be perceived as a greater influence on sleep quality than T1DM: “*I do not think so. I think that stress, yes, but diabetes per se, no. Well, if you have a hypo* [hypoglycemia] *or a hyper* [hyperglycemia] *it alters you a little, right? But, in general terms, to answer you in a broad sense, no.”* (E32, Man 27, HbA1c 55 mmo/mol (7.2%), CSII). Likewise, the lack of awareness also contributes to ignore the influence of diabetes on sleep quality: *“I do not think it does. Maybe it does, but I'm not sure.”* (E59, Woman 20, HbA1c 55 mmol/mol (7.2%), MDI). This lack of awareness is present even when there are several glycemic alterations overnight and negative effects: “*I do not know, I have not thought about it. I wasn't aware of the relation sleep-diabetes. Sometimes I'm aware of nightmares which I associate to hypoglycemias, but never thought that a rise* [hyperglycemia] *may affect getting back to sleep, or a drop* [hypoglycemia]*… I have not thought about it, to be honest.”* (E51, Woman 26, HbA1c 50 mmol/mol (6.7%), CSII).

## 4. Discussion

The purpose of this study is to explore the perceptions of emerging adults in Andalusia on the influence of T1DM on their sleep quality, considering the frequency of nocturnal glycemic alterations that cause awakenings. Results categorized participants into two groups: those who perceive this influence and those who do not. The former group accounts for slightly more than one-third of the participants (38%), with nearly all of these (92.6%) acknowledging negative personal, social, academic, or work consequences the day after experiencing an overnight glycemic alteration. While this perception is notably common among those experiencing three or more awakenings due to nocturnal glycemic alterations, it is also observed in those with a lower frequency of nocturnal glycemic alterations. On the other hand, more than half of our participants (62%) do not perceive the influence of T1DM on sleep quality, even though almost half of them (40.9%) experience three or more overnight glycemic alterations per week. Interestingly, compared to the first group, a larger proportion of these individuals (45.5%) do not acknowledge the negative impacts the following day. Despite 40% of participants who overlook these effects experiencing three or more overnight glycemic alterations, they regard the restoration of glycemic stability as merely another part of their routine care. They do not consider the challenge of returning to sleep significant, underscoring other factors such as stress as having a more substantial impact on sleep quality. Sleep patterns are highly susceptible to suffer alterations during emerging adulthood [1, 25]. As an aggravating circumstance, people with T1DM sleep less hours and suffer more sleep disorders, which worsen sleep quality in emerging adults with T1DM [10].

In this study, 62% of our participants did not perceive the influence of T1DM on their sleep quality, though 40.9% of them experienced three or more overnight glycemic alterations per week. Contrary to the results obtained by Griggs et al. [17], where all emerging adults expressed the influence of T1DM on their sleep, less than half of the participants (38%) in our study perceived the influence of diabetes on sleep quality. Regardless of whether they perceived the influence of T1DM, more than a third of our participants (38%) indicated some difficulty in getting back to sleep after an overnight glycemic alteration. This difficulty is also reflected in the study by Carreon et al. [19], which includes testimonies from children, adolescents, and adults with T1DM. These observations from our study are particularly significant considering that sleep quality, objectively assessed following the criteria of the National Sleep Foundation through medical diabetes devices, is associated with glycemic variability, with higher glycemic variability overnight being associated with worse sleep quality [26].

Almost all participants perceiving the influence of T1DM on sleep quality (92.6%) and more than half of those who do not perceive this influence (54.6%) described negative effects at academic, work, social, or personal level the day after experiencing an overnight glycemic alteration. Among the effects mentioned there is tiredness, worse performance at sport, academic, or work levels, and even absence from work or study center. These effects also coincide with the ones expressed by emerging adults with T1DM participating in the studies by Saylor, Hanna, and Calamaro [27] and Datye et al. [28]. Other effects described by this population in the literature are the difficulty for decision-making regarding diabetes, the effect on glycemic levels the following day, and the difficulty of self-management [27, 28]. In Griggs et al. [17] qualitative research, emerging adults with T1DM indicated general and specific barriers in relation to sleep and diabetes. According to this, interviewees in our study indicated specific barriers such as overnight glycemic alterations and diabetes device alarms, as well as broader issues, for instance, stress. The alarm of diabetes devices which help to manage the disease is a disruptor element mentioned by our participants and acknowledged in other studies [9, 18, 19].

More than half of our participants (62%) do not perceive the influence of T1DM on sleep, though among these, 40.9% may experience three or more overnight glycemic alterations per week, and 29.5% declared having a sleep interruption due to those alterations for thirty min or more. In the study by Griggs et al. [17], the key factor to perceive the influence of T1DM on sleep is the awakening caused by an overnight glycemic alteration. Contrarily, this circumstance does not determine the perception of the influence of diabetes on sleep quality among our participants.

Normalization of overnight glycemic alterations as everydayness may explain the high percentage of participants who, despite suffering negative effects, do not perceive the influence of T1DM on sleep quality. Thus, perception of glycemic management overnight may be distorted, as sometimes to perceive this influence is necessary that aggravating circumstances appear concomitant with the overnight hypoglycemia or hyperglycemia episodes, disregarding an area of improvement for both glycemic stability and quality of life. According to our participants and the emerging adults with T1DM involved in qualitative study of Datye et al. [28], some of these aggravating circumstances are repetitive episodes of hypoglycemia or hyperglycemia during the same night, overnight glycemic alterations prolonged over time, those episodes difficult to stabilize, and the ones occurring in the early hours.

In our study, we observed that there are emerging adults with T1DM who are not even aware of the relationship between diabetes and sleep quality, and therefore, they are unaware of the consequences implied. This finding contrasts with the results of the qualitative study by Saylor, Hanna, and Calamaro [27], where emerging adults with T1DM acknowledged the diabetes–sleep relationship and expressed that adequate sleep is a priority healthy habit for them. Our observation has not been reflected in other qualitative research on emerging adults with T1DM [17, 18, 27].

Findings from the current research have important clinical implications. Health professionals must consider the possibility of overnight glycemic alterations as an underlying cause of sleep disorders, thereby contributing to the prevention of normalizing glycemic instability that cause awakenings. Detection and treatment of hypoglycemia and hyperglycemia episodes overnight may improve both glycemic outcomes and sleep quality, thus helping to reduce complications associated to diabetes, poor sleep, and the high level of mortality associated [29]. Even when individuals do not express any concerns, it is crucial to assess them on sleep patterns, offering them different resources and informing them on the relation diabetes–sleep. This research underscores the urgency for health policymakers to prioritize sleep management in the care of people with diabetes. Policymakers should consider these findings when designing health campaigns, emphasizing the importance of sleep management in individuals with T1DM.

### 4.1. Strengths and Limitations

The main strength of this study is its large sample size, which enables a representation of the entire region of Andalusia, and simultaneously, it reinforces the reliability of the results. An additional strength is the use of cut-off points based on the DSM-5 diagnostic criteria for sleep disorders. This consideration allowed us to apply a clinically relevant framework to the categorization of the frequency of overnight glycemic alterations that cause awakenings observed in our participants, thereby enhancing the applicability of our findings. These results have not been described so far in other qualitative research studies [17, 18, 27]. This lack of awareness may be due to the normalization of the situation, the need of aggravating factors related to overnight glycemic alterations, or the lack of knowledge about healthy sleep [10, 18].

A notable aspect of this study lies in its focus on an age group that has received limited attention in previous research. Although the saturation point was reached with interview number 27, these interviews were only conducted in two of the eight provinces of Andalusia. Furthermore, at this point, more than half of the participants did not perceive that diabetes influences sleep quality, contrary to what has been reported in other studies [17, 19]. Therefore, the authors decided to conduct additional interviews to gather participants from all provinces of Andalusia and confirm that this percentage was not a chance result underscoring our commitment to have all necessary elements needed to build a comprehensive and conclusive theory [30].

This study sets a benchmark for rigorous research methodology by strictly adhering to the relevant COREQ (EQUATOR guidelines), ensuring that the findings are both credible and replicable in diverse settings. To ensure trustworthiness of our findings, we employed a triangulation process and had two authors independently code the initial interviews to establish emergent and superordinate themes. Additionally, in a subsequent individual interview, three participants verified the themes after these were explained and exemplified. These participants accepted the feedback and agreed to the established themes, thus reinforcing data credibility.

Regarding the limitations associated with qualitative methods and measures, the aforementioned strategy, concerning the process where the themes were verified by three participants, enhances the validity of our analysis. The iterative nature of qualitative analysis, its reliance on subjective interpretations, and the potential for researcher bias underscore the difficulty in achieving complete reliability and validity. Furthermore, the interviewer's background facilitated rapport but might have influenced analyses, which is why an independent coder was employed to analyze the initial interviews separately. Moreover, the use of purposive and subsequently snowball sampling may have introduced selection bias. This technique likely resulted in a sample that does not fully capture the demographic diversity or the differences between urban and rural settings among emerging adults with T1DM, along with their differing healthcare experiences. The implications of this method for the findings of this study suggest caution in the transferability of results to broader contexts.

In summary, although this study provides insightful perspectives on sleep quality among emerging adults with T1DM, the inherent limitations of the qualitative methods employed and the efforts to ensure trustworthiness require careful consideration. Future research would benefit from a broader array of qualitative measures, such as participant observation, diverse recruitment strategies, and further methodological enhancements. Lastly, though interviews revealed perceptions of the influence of T1DM on sleep quality, further qualitative longitudinal research is necessary for a deeper understanding of this relation and to assess changes over time.

## 5. Conclusion

Perception of the influence of T1DM on sleep quality not always coincides with a given frequency of awakenings generated by overnight hypoglycemias or hyperglycemias. Therefore, it could be assumed that the lack of perception is not always indicative of the absence of interference of T1DM on sleep. This lack of perception may be due to the unawareness, nonexistence of aggravating factors related to overnight glycemia alterations, or normalization of the situation. All these factors would hinder the detection of the glycemic alterations during the night, thus perpetuating glycemic imbalance during this age period and affecting sleep quality.

## Figures and Tables

**Table 1:** Superordinate and emergent themes.

1.	Perception of sleep
1.1	Sleep quality and diabetes
2.	Overnight glycemic management: hypoglycemia
2.1	Frequency of overnight hypoglycemias
2.2	Difficulty getting back to sleep
2.3	Span of sleep interruption
3.	Overnight glycemic management: hyperglycemia
3.1	Frequency of overnight hyperglycemia
3.2	Difficulty getting back to sleep
3.3	Span of sleep interruption
4.	Consequences of overnight glycemic alterations
4.1	Consequences regarding glycemia stability and insulin resistance
4.2	Consequences at functional and personal performance levels
4.3	Consequences at emotional, affective, and mood-related levels

**Table 2:** Participants' characteristics (*n* = 71).

**Demographic features**	**Median (** **n** **)**	**IQR (%)**
*Age*	20	5
18–25	59	83.1%
26–29	12	16.9%
*Biological sex*		
Male	30	42.3%
Female	41	57.7%
*Ethnicity*		
Andalusians-Hispanic	71	100%
*Body mass index* ^a^	23.5	3.1
Normal weight (18.5–24.9)	54	76%
Overweight (25–29.9)	13	4.2%
Class 1 obesity (30–34.9)	3	4.2%
Class 2 obesity (35–39.9)	1	1.4%
*Geographical location*		
Almeria	4	5.6%
Cadiz	10	14.1%
Cordoba	6	8.5%
Granada	6	8.5%
Huelva	2	2.8%
Jaen	10	14.1%
Malaga	7	9.9%
Seville	26	36.6%
*Finished education*		
Compulsory secondary-school education	4	5.6%
High school	8	11.3%
Vocational education	13	18.3%
University degree	39	54.9%
Master or postgraduate	7	9.9%
*Employment status*		
Full-time work	15	21.1%
Part-time work	2	2.8%
Casual/temporary work	6	8.5%
Works in no-class periods	2	2.8%
Studies and works	13	18.3%
Does not work	33	46.5%
*Living arrangements*		
With parents/legal tutors	51	71.8%
With partner	5	7%
Alone	3	4.2%
With other relatives	2	2.8%
With roommates	8	11.3%
University residence hall	2	2.8%

^a^World Health Organization classification of adults according to body mass index.

**Table 3:** Clinical features.

**Clinical features**	**Median (** **n** **)**	**IQR (%)**
*Type 1 diabetes mellitus profile*		
T1DM duration (years)	11	10
Hemoglobin A1c^a^	7.2 55.68 mmol/mol	0.9 6.96 mmol/mol
< 53 mmol/mol, < 7%	25	35.2%
≥ 53 mmol/mol, ≥ 7%	46	65.8%
*Treatment*		
MDI (%yes)	52	73.2%
CSII (%yes)	19	26.8%
CGM/flash system (%yes)	66	93%

Abbreviations: CGM/flash system: continuous glucose monitoring/flash glucose monitoring system; CSII: continuous subcutaneous insulin infusion; MDI: multiple daily injections; T1DM: Type 1 diabetes mellitus.

^a^HbA1c is reported in IFCC units (mmol/mol) and NGSP units.

**Table 4:** Perception of the influence of Type 1 diabetes mellitus on sleep quality.

**Perception of the influence of T1DM on sleep quality**	**Perception of the frequency of self-reported influence**	**Number of participants with ≤ 2 or > 2/overnight glycemic alterations per week**		**Perception of negative effects the following day**		**Difficulty in getting back to sleep**		**Interruption span**	
				**After an overnight glycemic alteration**					
				**Yes**	**No**	**Yes**	**No**	**< 30** **min**	**≥ 30 min**
**Yes: There is influence**	**Many times**	**≤ 2/week**	6	6	0	4	2	1	5
		**> 2/week**	6	6	0	4	2	5	1
	**Sometimes**	**≤ 2/week**	5	3	2	4	1	3	2
		**> 2/week**	10	10	0	1	9	6	4

**No: There is no influence**	**Rarely**	**≤ 2/week**	3	2	1	1	2	2	1
		**> 2/week**	8	4	4	2	6	3	5
	**Never**	**≤ 2/week**	23	12	11	9	14	18	5
		**> 2/week**	10	6	4	2	8	8	2

Abbreviations: > 2: more than two glycemic alterations per week; ≤ 2/week: equal or less than two overnight glycemic alterations per week; < 30 min: less than 30 min; ≥ 30 min: equal or more than 30 min; T1DM: Type 1 diabetes mellitus.

## Data Availability

Due to the sensitive nature of the questions asked in this study, respondents were assured that raw data would remain confidential and would not be shared openly. The audio and transcript interviews are securely archived in a folder under the custody of the researcher who conducted these interviews: MANB. However, anonymized or aggregated data are available from the first author (mnbaila@us.es) upon reasonable request, ensuring no identification of the participants is possible.
