# Supplementary material for: Perceptions of Emerging Adults With Type 1 Diabetes Mellitus on How the Condition Influences Sleep Quality: A Qualitative Study
Source: J Diabetes Res. 2024 Jul 10;2024:7497059. doi: 10.1155/2024/7497059 (PMC11254458; doi:10.1155/2024/7497059)
Supplement: Supporting Information — Additional supporting information can be found online in the Supporting Information section. Interview script. [file 7497059.f1.docx]

**SUPPLEMENTARY MATERIAL**

**INTERVIEW SCRIPT**

1. Existence or absence of perception regarding the influence of type 1 diabetes mellitus on sleep quality

2. Justification and reasons for perceiving or not this influence

3. Overnight glycemic alterations: Hypoglycemia

3.1. Frequency of nocturnal hypoglycemia episodes per week

3.2. Perception of the relevance and impact of this frequency on sleep quality

3.3. Time required to get back to sleep after an episode of nocturnal hypoglycemia

3.4. Perception of the episode of nocturnal hypoglycemia duration as relevant

3.5. Side effects and consequences

3.6. Difficulty in getting back to sleep

4. Overnight glycemic alterations: Hyperglycemia

4.1. Frequency of nocturnal hyperglycemia episodes per week

4.2. Perception of the relevance and impact of this frequency on sleep quality

4.3. Time required to get back to sleep after an episode of nocturnal hyperglycemia

4.4. Perception of the episode of nocturnal hyperglycemia duration as relevant

4.5. Side effects and consequences

4.6. Difficulty in getting back to sleep
